# Supplementary material for: MetaQTL: a package of new computational methods for the meta-analysis of QTL mapping experiments
Source: BMC Bioinformatics. 2007 Feb 8;8:49. doi: 10.1186/1471-2105-8-49 (PMC1808479; doi:10.1186/1471-2105-8-49)
Supplement: Additional File 7 — MetaQTL Package : jar file and tutorial. This Zip archive contains both the MetaQTL JAR file and the files of the tutorial. [file 1471-2105-8-49-S7.zip › org.inra.metaqtl/doc/org/metaqtl/bio/entity/factory/XmlGeneticMapFactory.html]

XmlGeneticMapFactory


|  |  |  |
| --- | --- | --- |
| **Overview** \| **Package** \| **Class** \| **Use** \| **Tree** \| **Deprecated** \| **Index** \| **Help** | |  |
| **PREV CLASS**   **NEXT CLASS** | **FRAMES**    **NO FRAMES**     **All Classes** |  |
| SUMMARY: NESTED \| FIELD \| CONSTR \| METHOD | DETAIL: FIELD \| CONSTR \| METHOD |  |


---


## org.metaqtl.bio.entity.factory Class XmlGeneticMapFactory

```
java.lang.Object
  org.metaqtl.bio.entity.factory.BioEntityFactory
      org.metaqtl.bio.entity.factory.XmlBioEntityFactory
          org.metaqtl.bio.entity.factory.XmlGeneticMapFactory
```

---

``` public class XmlGeneticMapFactory extends XmlBioEntityFactory ```

Class Description Here

**Author:**
:   Jean-Baptiste Veyrieras

---

**Constructor Summary**

`XmlGeneticMapFactory()`


| **Method Summary** | |
| --- | --- |
| `protected  java.lang.Class` | `getEntityBeanClass()` |
| `protected  int` | `getEntityType()` |

**Methods inherited from class org.metaqtl.bio.entity.factory.XmlBioEntityFactory**

`load, load, unload, unload`

**Methods inherited from class java.lang.Object**

`clone, equals, finalize, getClass, hashCode, notify, notifyAll, toString, wait, wait, wait`

**Constructor Detail**

### XmlGeneticMapFactory

```
public XmlGeneticMapFactory()
```


**Method Detail**

### getEntityBeanClass

```
protected java.lang.Class getEntityBeanClass()
```

:   **Specified by:**: `getEntityBeanClass` in class `XmlBioEntityFactory`

---


### getEntityType

```
protected int getEntityType()
```

:   **Specified by:**: `getEntityType` in class `XmlBioEntityFactory`


---


|  |  |  |
| --- | --- | --- |
| **Overview** \| **Package** \| **Class** \| **Use** \| **Tree** \| **Deprecated** \| **Index** \| **Help** | |  |
| **PREV CLASS**   **NEXT CLASS** | **FRAMES**    **NO FRAMES**     **All Classes** |  |
| SUMMARY: NESTED \| FIELD \| CONSTR \| METHOD | DETAIL: FIELD \| CONSTR \| METHOD |  |


---
